# Self-Stigma Among People With Mental Health Problems in Terms of Warmth and Competence

**DOI:** 10.3389/fpsyg.2022.877491

**Published:** 2022-06-14

**Authors:** Laura Gärtner, Frank Asbrock, Frank Euteneuer, Winfried Rief, Stefan Salzmann

**Affiliations:** ^1^Department of Clinical Psychology and Psychotherapy, Philipps University of Marburg, Marburg, Germany; ^2^Department of Psychology, Chemnitz University of Technology, Chemnitz, Germany; ^3^Department of Psychology, Clinical Psychology and Psychotherapy, Medical School of Berlin, Berlin, Germany

**Keywords:** self-stigma, stigma, stereotype content model, BIAS map, warmth, competence, mental health

## Abstract

**Introduction:**

Self-stigma arising from public stigma is a heavy burden for people suffering from mental health problems. Both public stigma and self-stigma encompass the same three elements: stereotype, prejudice, and discrimination. Public stigma has already been successfully explored by the Stereotype Content Model (SCM) and the Behaviors from Intergroup Affect and Stereotypes (BIAS) map. However, this is not the case for self-stigma. Therefore, this is the first study that applies SCM and the BIAS map to self-stigma by examining whether the effects of self-stereotypes on self-directed discrimination would be mediated by self-directed prejudices in people with mental health problems.

**Method:**

Within a total sample of *N* = 823 participants, who took part in an online survey, *n* = 336 people reported mental health problems. Mental health and self-stereotypes (warmth, competence), self-directed prejudice (negative emotions), and self-directed discrimination (active/passive self-harm) were assessed.

**Results:**

Structural equation modeling supported the hypothesis that the stereotype dimensions warmth and competence negatively related to prejudice, while stronger prejudice was associated with more discrimination (active/passive self-harm). Prejudice fully mediated the relationship between stereotypes and discrimination. The indirect effects of warmth and competence on active and passive self-harm were moderated by competence and warmth.

**Discussion:**

Implications for further research on self-stigma and the usage of SCM and BIAS map are discussed.

## Introduction

Mental health problems are challenging our societies. It is estimated that, approximately, 165 million people suffer from mental disorders in Europe. This corresponds to 12-month prevalence of 38.2% (Wittchen et al., 2011) and causes costs of over EUR 600 billion (OECD, 2018). Those affected have to carry a double burden. They not only suffer from disease-specific symptoms, functional disabilities (Buist-Bouwman et al., 2006; Mack et al., 2015), or decreased quality of life (Alonso et al., 2004a; Mack et al., 2015) but also public stigma (Rüsch et al., 2005). Public stigma is an additional pitfall because it is associated with self-stigma (Vogel et al., 2013). It is assumed that public stigma and, especially, self-stigma are barriers to help seeking behavior (Rüsch et al., 2005; Clement et al., 2015; Cheng et al., 2018; Schomerus et al., 2019). Since only 25.7% of those who met the criteria for a mental disorder in the past 12 months are using mental health services (Alonso et al., 2004b), a better understanding of the components of self-stigma and their interaction is needed to bridge the gap for better health care. Since the Stereotype Content Model (SCM, Fiske et al., 2002) and the Behaviors from Intergroup Affect and Stereotypes (BIAS) map (Cuddy et al., 2007) are well-established theories that were already successfully applied to public stigma, it seems obvious to apply them to self-stigma as well so that both—public stigma and self-stigma—can be integrated within the same theoretical framework. This is the first study to examine whether the SCM and the BIAS map can be applied to self-stigma.

### Public Stigma

Public stigma is described as society's negative reaction toward people with mental illness (Corrigan and Watson, 2002a). People with mental illness are strongly affected by public stigma and experience even more stigma than people with physical disabilities (Kowalski and Peipert, 2019). Due to the high prevalence of mental illness stigma (Tzouvara et al., 2016), the consequences are far-reaching, for example, reduced use of mental health services (Schomerus et al., 2019), reduced mental health (Ilic et al., 2013), or dehumanization of people with mental illness (Boysen et al., 2020a,b). Based on the *social-cognitive model of public stigma* (Corrigan, 2000; Corrigan and Watson, 2002a), it is assumed that public stigma encompasses three elements: stereotypes, prejudice, and discrimination. Stereotypes are shared beliefs about groups, generalizing the characteristics of members and neglecting differences among them (Aronson et al., 2014). Common stereotypes about people with mental illness address dangerousness and reduced competence (Crisp et al., 2000; Parcesepe and Cabassa, 2013). Prejudices are negative emotional evaluations. Examples of prejudice against people with mental illness are fear, anger, and pity (Corrigan et al., 2003; Angermeyer et al., 2010). Discrimination as negative group-based behavior against people with mental illness includes withholding help (Corrigan, 2000), not hiring someone (Corrigan and Watson, 2002a; Tzouvara et al., 2016), rejection from potential mates (Boysen et al., 2019), and social distancing (Parcesepe and Cabassa, 2013). The elements of public stigma are linked since stereotypes are associated with prejudices, which are associated with discriminatory behavior (Corrigan and Watson, 2002a). Discrimination against people with mental illness varies with the valence and severity of stereotypes and prejudices (Corrigan and Watson, 2002a). Applying the Stereotype Content Model framework (Fiske et al., 2002; Cuddy et al., 2007), Sadler et al. (2015) showed, for example, that mental illness stereotypes correlated with specific emotional and behavioral action tendencies. The Stereotype Content Model is a universal model for group perception, which we applied for analyzing the self-stigma of people with mental health problems in the present study.

### Stereotype Content Model and Behaviors From Intergroup Affect and Stereotypes Map

According to the Stereotype Content Model (SCM; Fiske et al., 2002; Fiske, 2018), stereotypes boil down to two fundamental dimensions: warmth (W) and competence (C). Warmth represents the intention of group members, ranging from competition [cold/low warmth (LW)] to cooperation [warm/high warmth (HW)]. Competence represents the ability of group members to achieve their goals, which is related to low [incompetent/low competence (LC)] or high status [competent/high competence (HC)]. Out of the interaction of warmth and competence arise four types of stereotype content: First, groups perceived as warm but incompetent (HW/LC), for example, older people (Durante et al., 2013) and people with physical disabilities (Meyer and Asbrock, 2018). They provoke mostly pity and sympathy. Second, groups perceived as competent but cold (LW/HC), for example, rich people (Durante et al., 2013; Meyer and Asbrock, 2018) or feminists (Fiske et al., 2002; Durante et al., 2013). They provoke envy and jealousy. Third, groups perceived as warm and competent (HW/HC), for example, in-group or middle-class (Fiske et al., 2002; Durante et al., 2013). They provoke admiration and pride. Fourth, groups perceived as cold and incompetent (LW/LC), for example, homeless people (Lee and Fiske, 2006), or people with mental illness (Sadler et al., 2012; Meyer and Asbrock, 2018; Boysen et al., 2020a). They provoke contempt, disgust, and anger. Within the group of people with mental illness, stereotype content varies between different disorders. Those with schizophrenia, multiple personality disorders, or addictions are, for example, perceived as especially low in warmth and competence (Fiske, 2012; Sadler et al., 2012). The SCM is a highly established model and has been proved to be stable across cultures and countries (Cuddy et al., 2009; Asbrock, 2010; Durante et al., 2013; Bye et al., 2014; Fiske, 2018), while there are also cultural variations, for example, depending on a society's extent of equality or individuality (Fiske and Durante, 2016). In addition, the SCM is applicable to different levels of social evaluation: intergroup, interpersonal, and individual levels (Cuddy et al., 2008; Russell and Fiske, 2008; Aragonés et al., 2015).

The Behaviors from Intergroup Affect and Stereotypes (BIAS) map (Cuddy et al., 2007) extends the SCM and differentiates four types of behavior tendencies based on perceived warmth, competence, and the associated emotions. Warmth predicts active behaviors: active facilitation (e.g., help, protect) toward others perceived as warm and active harm (e.g., fight, attack) toward those perceived as cold. Competence predicts passive behaviors: passive facilitation (e.g., cooperate with) toward others perceived as competent and passive harm (e.g., exclude, ignore) toward those perceived as incompetent. Emotions have particular importance within the BIAS map because they predict behavior tendencies more strongly and directly than warmth and competence: emotions mediate the relationship between stereotypes and behaviors. Admiration (HW/HC) predicts active and passive facilitation, contempt (LW/LC) predicts active and passive harm, pity (HW/LC) predicts active facilitation and passive harm, and envy (LW/HC) predicts active harm and passive facilitation (Cuddy et al., 2007; Echebarria-Echabe, 2013; Key et al., 2019; Constantin and Cuadrado, 2020; Findor et al., 2020).

### Stereotype Content Model, BIAS Map, and Public Stigma

The SCM and the BIAS map have been used to examine public stigma of people with mental illness (Sadler et al., 2015; Iles et al., 2016; Thonon et al., 2016; Boysen, 2017). This is due to the advantage of the SCM and the BIAS map to specify the prediction of specific stereotypes on specific emotions, and specific behavioral tendencies as well as their interactions. Meaning that perceived warmth and competence of people with mental illness is predicting emotional prejudice like contempt (Sadler et al., 2015; Iles et al., 2016), as well as discrimination like active and passive harm (Boysen, 2017). Sadler et al. (2015) examined public stigma for different subgroups of people with mental illness and found support for the mediating relationship of emotions between stereotypes and discriminatory behavior. Furthermore, they observed anger and fear as different emotions outside of contempt (LW/LC) because of their unique position in public stigma literature. This is due to the observation that stigmatized people are frequently confronted with someone's anger and fear (Corrigan et al., 2003; Angermeyer et al., 2010). Anger and fear are primarily predicted by warmth (Boysen, 2017) and, therefore, should lead to active behavior (Cuddy et al., 2007). Following this, Sadler et al. (2015) could show that anger mediated the relation between warmth and active harm. On the contrary, fear was predicted by both warmth and competence, with the result that fear mediates the association between both stereotype dimensions and passive harm (Sadler et al., 2015). In line with this, emotional prejudice, involving anger and fear besides contempt, occupies a central position within public stigma.

### Self-Stigma

Self-stigma of mental illness is described as the internalization of negative stereotypes from a society that broadly endorses stigmatization (Corrigan and Watson, 2002a,b). Those stereotypes may address incompetence or dangerousness (Corrigan and Rao, 2012). Self-stigma is not only associated with public stigma (Vogel et al., 2013) but also encompasses the same three elements: stereotype, prejudice, and discrimination. However, the elements of self-stigma refer to oneself and not to others (Corrigan and Watson, 2002a). People with mental illness are aware of stereotypes that concern people like them (e.g., “*Mentally ill persons are incompetent*.”). If they agree to them and apply them to the self, harming cognitions ensue, and the process of internalization is completed (e.g., “*I'm incompetent and not worthy.”*; (Corrigan et al., 2011; Corrigan and Rao, 2012). These stereotypes can result in self-prejudice, which is conceptualized as a negative affective reaction toward the self (Corrigan and Watson, 2002b), including emotions like fear (Corrigan and Rao, 2012) or shame (Kranke et al., 2010; Rüsch et al., 2010; Hasson-Ohayon et al., 2012; Tucker et al., 2013; Birtel et al., 2017). In addition to this, self-prejudice is also strongly related to low self-esteem, which, again, is associated with negative affective reactions like shame (Budiarto and Helmi, 2021), depression or anxiety (Sowislo and Orth, 2013). Negative affective reactions, in turn, can elicit discriminatory behaviors against the self (e.g., self-isolation Corrigan and Rao, 2012) or secrecy of the mental illness (Stolzenburg et al., 2017). Self-stigma is associated with several negative outcomes, such as decreased quality of life (Corrigan and Rao, 2012; Kao et al., 2016) and well-being (Kao et al., 2016; Rose et al., 2019), reduced help-seeking behavior (Evans-Lacko et al., 2012) or non-adherence to or drop-out from treatment (Corrigan et al., 2014), as well as increased symptom severity (Boyd et al., 2014), depressiveness (Kao et al., 2016), and suicidality (Oexle et al., 2018). Even though self-stigma and a negatively biased view of the self as a symptom of depression overlap at some point, it is important to distinguish them. First, self-stigma is prevalent among various mental disorders besides depression like schizophrenia spectrum disorder, other mood disorders, anxiety disorders, PTSD, borderline personality disorder, autism spectrum disorder, and eating disorders (Griffiths et al., 2015; Bonfils et al., 2018; Dubreucq et al., 2020). Second, not all individuals with depression experience self-stigma. Only one in five people with depression or bipolar disorder suffers from a moderate to a high level of self-stigma (Brohan et al., 2011). At last, self-stigma is, instead, an additional burden for those who suffer from depression and, therefore, should be addressed on its own when trying to improve shared negative outcomes like low self-esteem or avoidance (Corrigan et al., 2006; Manos et al., 2009; Shimotsu and Horikawa, 2016).

### The Present Study

People with mental illness are confronted with a double burden: On the one hand, they suffer from disease-specific symptoms and functional disabilities (Buist-Bouwman et al., 2006; Mack et al., 2015). On the other hand, they experience public and self-stigma with negative consequences (Rüsch et al., 2005). While previous research applied the SCM (Fiske et al., 2002) and the BIAS map (Cuddy et al., 2007) to public stigma of people with mental illnesses (Sadler et al., 2015; Iles et al., 2016; Thonon et al., 2016; Boysen, 2017), to the best of our knowledge, self-stigma has not been analyzed in this theoretical context. These well-established models, however, are likely to provide a systematic framework for understanding the specific relations between self-stereotyping, self-prejudice, and self-directed discrimination. This assumption receives support from the *Dual Perspective Model of Agency and Communion* [DPM-AC (Abele and Wojciszke, 2014; Abele et al., 2021)]. It also refers to two fundamental dimensions similar to warmth and competence (communion and agency). It provides a broader empirical endorsement for the applicability of those two dimensions within an intraindividual context like self-stigma. However, it is still uncertain if SCM and the BIAS map are suitable to make predictions for self-stigma and whether the effects of stereotypes on discrimination are also mediated by prejudice within self-stigma. Thus, this study aimed to apply the specific predictions of the SCM and the BIAS map to self-stigma among people with mental health problems. Since self-stigma as internalization of negative stereotypes includes harmful cognitive and emotional reactions toward the self, the resulting behavioral reactions typically have a negative valence (Corrigan, 2016). In terms of the SCM and the BIAS map, the internalization of negative stereotypes, respectively, harmful cognitions, is represented by low warmth and low competence. These predict contempt as an emotional reaction and active as well as passive harm as a behavioral reaction. Therefore, we focused our hypotheses on active and passive (self-) harming behavioral tendencies, meaning that they result in a negative outcome for the affected one (the self). (Self-)Prejudice in the context of self-stigma contains several negative emotional reactions (e.g., fear, shame), which are most likely allocated in the same cluster—low warmth/low competence—as contempt. It was expected that self-directed stereotypes (warmth, competence) of people with mental health problems predict self-prejudice (negative emotions), as well as discrimination (active and passive self-harm). Negative emotions were hypothesized to be associated with active and passive self-harming behavior and to mediate the relationship between warmth and active self-harm, as well as competence and passive self-harm. The interaction of warmth and competence was assumed to influence negative emotions as well as active and passive self-harm. Verifying the use of SCM and the BIAS map to explain self-stigma would allow a stronger theoretical link between public and self-stigma. Furthermore, the universal theoretical framework could help researchers explain self-stigma for all kinds of mental health problems, physical disease, and even for other reasons for self-stigma like sexual orientation. A better understanding of self-stigma could also help to shed more light on specific mechanisms, which may be essential to developing effective interventions to reduce self-stigma.

## Materials and Methods

### Participants

Three-hundred and thirty-six predominant female (73.2%) individuals aged 18 to 62 (*M* = 26.79, *SD* = 9.44), with mental health problems (clinical subsample A), as well as 393 healthy individuals (healthy subsample B; 74.8% female, aged 18–76, *M*_age_ = 27.34, *SD*_age_ = 10.39) within a total sample of *N* = 823 participants (74.4% female, aged 18–76, *M*_age_ = 28.15, *SD*_age_ = 11.16), were recruited through University mailing lists, social media, as well as flyers at public places between November 2019 and June 2020. Individuals with mental health problems (A) reported to have mental health problems (*n* = 145), a diagnosed mental disorder (*n* = 98) or passed at least one cut-off score, which indicates a mental disorder within the German Patient Health Questionnaire (PHQ-D; *n* = 277). Healthy participants (B) reported to have neither mental nor physical health problems or disease and passed no cut-off score of the PHQ-D. Most of the participants were students (*n* = 543, 66.0%) or employed (*n* = 218, 26.5%). Participation was voluntary in return for course credit or the chance to win one of five gift cards (25€). The study was approved by the Local Ethics Committee. All the participants gave informed consent prior to participation. Detailed sociodemographic and clinical characteristics for individuals with mental health problems (A), as well as healthy participants (B), are presented in Table 1.

**Table 1:** Sociodemographic and clinical characteristics of the subsamples A (*n* = 336) and B (*n* = 393).

	**A** **Individuals with mental health problems**		**B** **Healthy individuals**	
**Characteristic**	***n*** **or Mean**	**% or *SD***	***n*** **or Mean**	**% or *SD***
**Gender**
Male	83	(24.7)	92	(23.4)
Female	246	(73.2)	294	(74.8)
Diverse	4	(1.2)	2	(0.5)
**Age**	26.79	(9.44)	27.34	(10.39)
**Highest educational level**
Middle school (10th grade)	16	(4.8)	9	(4.8)
High school	214	(63.7)	230	(63.7)
University or postgraduate degree	87	(25.9)	139	(25.9)
Others	17	(5.1)	8	(5.1)
**Employment**
Unemployed	4	(1.2)	1	(1.2)
Employed	69	(21.1)	105	(21.1)
Student	241	(71.7)	262	(71.7)
Retired	2	(0.6)	2	(0.6)
Others	18	(6.4)	15	(6.4)
**Self-reported diagnosis**
F1 (Abuse, Addictions)	3	(0.9)		
F2 (Schizophrenia)	2	(0.6)		
F3 (Affective disorders)	50	(14.9)		
F40, F41 (Anxiety disorders)	45	(13.4)		
F42 (Obsessive-compulsive disorders)	11	(3.3)		
F43 (PTSD)	19	(5.7)		
F45 (Somatoform disorders)	5	(1.5)		
F5 (Eating disorders)	27	(8.0)		
F6 (Personality disorders)	17	(5.1)		
Others	5	(1.5)		
**PHQ-D: Clinical relevance**
Somatoform disorders	85	(25.3)		
Depressive disorders	92	(27.4)		
Anxiety disorders	83	(24.7)		
Eating disorders	23	(6.8)		
Alcohol disorders	137	(40.8)		

### Materials

#### Mental Health

Mental health was measured with the German Patient Health Questionnaire (PHQ-D; Löwe et al., 2002). The PHQ-D is based on the criteria from the Diagnostic and Statistical Manual of Mental Disorders - Fourth Edition (DSM–IV; American Psychiatric Association, 2000). It was used as a screening instrument for five common mental disorders (the participants were included if they scored above the respective cut-off scores). The PHQ-D has 78 items and allows for provisional diagnosing somatoform (Cronbach's α = 0.79; Gräfe et al., 2004), depressive (Cronbach's α = 0.88; Gräfe et al., 2004), anxiety (Cronbach's α = 0.89; Löwe et al., 2008), eating and alcohol disorder within independent modules. Cut-off scores were used as defined in the PHQ-D instruction manual (Löwe et al., 2002).

#### Stereotype

The stereotype dimensions warmth and competence were measured with three items each on a seven-point Likert scale (0 = *not at all* to 6 = *completely*). These items are based on Fiske et al. (2002) and were adopted from Eckes (2002) and Asbrock (2010). The frame of reference of all items was changed so that they focus on the self (*I see myself as […]*). Warmth was assessed by *likable, warm, good-natured*; competence by *independent, competitive*, and *competent*^1^. In the present study, the internal consistencies (Cronbach's α) for warmth and competence were 0.71 and 0.79, respectively.

#### Prejudice

In the context of self-stigma, prejudice is defined as negative emotional reaction toward the self (Corrigan and Watson, 2002b). Four items (*contempt, anger, fear*, and *shame*^2^) from the low warmth/low competence cluster were used to measure the frequency of experiencing negative emotions. Contempt was derived directly from the SCM (Fiske et al., 2002). Anger, fear, and shame were added because they are relevant not only in the context of public stigma (Angermeyer et al., 2010; Sadler et al., 2015; Birtel et al., 2017) but also self-stigma (Corrigan and Rao, 2012; Tucker et al., 2013; Pérez-Ramírez et al., 2021). In addition, anger and fear were already used in the context of the SCM and the BIAS map and predicted harming behavior (Cuddy et al., 2007; Sadler et al., 2015). The phrasing of the items refers to Fiske et al. (2002) and Cuddy et al. (2007). Prejudice was measured with four items on a seven-point Likert scale (0 = *never to* 6 = *very often*). An example was given for every item (*contempt: I'm feeling contempt for myself*; *anger: I'm angry with myself*; *fear: I'm afraid of myself*; *shame: I'm ashamed of myself*). Cronbach's α was 0.83 in this study.

#### Discrimination

Discrimination as behavioral tendencies resulting in negative outcomes for the self was distinguished in active and passive self-harm. Both were measured with three items each on a seven-point Likert scale (0 = *not at all* to 6 = *completely*). Sixteen items were initially used for assessing active and passive self-harm. Observing all items with an exploratory factor analysis (EFA) with promax rotation indicated a three-factor solution. One factor could be interpreted as active self-harm [*I injure myself* (active_sh_1)^3^, *I inflict bodily and mental pain on myself* (active_sh_2)*, I intentionally harm my body* (active_sh_3)] and is comparable to the concept of non-suicidal self-injury (NSSI), describing the intended injury of body tissue without the purpose to die (Nock, 2010; Petermann and Nitkowski, 2011; Zetterqvist, 2015). The second factor was interpreted as passive self-harm (seven items: *I distance myself from friends and colleges, I avoid to disclose something about me, I refuse invitations and appointments, I reject offers of help, I don't make new contacts to avoid rejections, I keep problems to myself to not be a burden to someone, I avoid to focus on my thoughts, feelings, and needs*). While conceptualizing passive self-harm for this study, we focused on combining the concepts of indirect self-injury (St Germain and Hooley, 2012) and indirect self-destructiveness (Kelley et al., 1985), with the definition of passive harm on an intergroup level (Cuddy et al., 2007). Indirect self-injury and indirect self-destructiveness describe behavior that increases the probability of future negative consequences and includes avoiding or omitting action while excluding injuring body tissue (Kelley et al., 1985; St Germain and Hooley, 2012; Tsirigotis, 2018). Passive harm on an intergroup level means to reduce social worth by ignoring, neglecting, or excluding others so that social recognition is omitted (Cuddy et al., 2007). Transferring this to passive self-harm means that we ascertain self-directed behavior, which is characterized by avoiding or omitting actions that would lead to social recognition, for example, avoiding to join social activities, or excluding oneself from others. A second EFA with promax rotation was performed for passive self-harm to reduce the scale up to three items as well [*I distance myself from friends and colleges* (passive_sh_1)*, I refuse invitations and appointments* (passive_sh_2), *I avoid to disclose something about me* (passive_sh_3)]. The third factor (8 items, e.g., *I keep telling myself that I'm worthless, I insult myself and swear at me, I tell myself that I'm incompetent*) was rejected because it did not meet the definition either of active nor passive self-harm. The internal consistencies (Cronbach's α) were 0.89 for active self-harm and 0.76 for passive self-harm.

### Procedure

The participants took part in an online survey on “Healthy or ill: When do we help others and ourselves? An online survey on health-related self- and external perception”^4^. To reduce socially desirable responses, we refrained from using stigma-associated words in the title and the whole survey. After providing sociodemographic information, the participants were asked for their mental and physical health status, including the question if they suffer from mental health problems and diagnosed mental disorders. They were also asked for the kind of mental disorder they suffer the most from. Additionally, the participants were screened for mental disorders using the PHQ-D. All the participants responded to items, measuring warmth, competence, negative emotions, and active and passive self-harm. They also filled in other questionnaires (e.g., public stigma, health-related quality of life) that are not reported here. After finishing the questionnaire, the participants were thanked for their participation.

### Statistical Analysis

Data preparation and descriptive statistics were performed using IBM SPSS statistics, Version 26. Missing data were examined for the total sample, which ranged from 0 to 0.6% per item. Little's MCAR test [χ^2^(73) = 65.003, *p* = 0.735] indicated that missing cases were completely missing at random. So, full information maximum likelihood (FIML) estimation was used to consider missing data. Items were screened for univariate (*z*-scores > 3.29) and multivariate outliers (mahalanobis distance, χ^2^, *p* < 0.001), and 22 cases of the total sample were excluded, reducing it to *N* = 801. All statistical analyses concerning the clinical subsample A were performed with *n* = 320 participants and *n* = 393 participants for the healthy subsample B, respectively. Structural equation models (SEM) were conducted in Mplus Version 7 (Muthen and Muthen, 2012) using FIML estimator. Bootstrapping (*N* = 5000) was applied due to the non-normality of the data. A model was specified in which the stereotype dimensions warmth and competence predict the discriminatory dimensions of active and passive self-harm. Prejudice was complemented as a mediator, following the theoretical implications of the SCM and the BIAS map. Latent variables were defined for all constructs. Warmth and competence, as well as active and passive self-harm, were allowed to correlate. Model modification indices were used to improve the model fit. To evaluate the model fit χ^2^, root mean square error of approximation (RMSEA), comparative fit index (CFI), Non-Normed Fit Index (TLI), and standardized root mean square residual (SRMR) were examined. RMSEA and SRMR values < 0.05 indicate good fit; values between 0.05 and 0.08 suggest a reasonable fit (Browne and Cudeck, 1993; Schreiber et al., 2006). A CFI likewise TLI value > 0.95 indicates a good fit; a value above 0.90 suggests acceptable fit (Hu and Bentler, 1999; Schreiber et al., 2006). Indirect effects were computed using bias-corrected bootstrap confidence intervals. Considering that different combinations of warmth and competence, mixed and consistent stereotypes, are associated with distinct emotional and behavioral reactions within the framework of the SCM and the BIAS map, we also examined how the interaction of warmth and competence affects prejudice and discrimination within self-stigma in a separate model. The previously described structural equation model was complemented by a latent interaction term of warmth and competence (warmth x competence) that predicted negative emotions, passive self-harm, and active self-harm. Conditional indirect effects were calculated. All variables were standardized. Besides, Akaike-Information-Criterion (AIC) and Bayesian-Information-Criterion (BIC), the log-likelihood values were compared. A log-likelihood ratio test was performed to contrast the latent interactive model with the initial model. A significant result of the log-likelihood ratio test would indicate a significant loss in the model fit of the initial model relative to the latent interactive model. Relatively lower values of AIC and BIC indicate better model fit.

#### Additional Analyses

Research has already shown that gender is an important attribute when evaluating others on the fundamental dimensions of warmth and competence within the SCM and the BIAS map. Thus, there are differences between genders regarding warmth and competence (Eckes, 2002; Fiske, 2010; Fiske and Durante, 2016). Considering this, gender could also be meaningful when it comes to self-evaluation and self-stigma. Additional analyses were performed to examine the potential influence of gender (1 = women, 2 = men) on self-stereotypes, self-prejudice, and self-discrimination. Because of the small number (*n* = 4) of individuals, who stated their gender as diverse, they were not included in the analyses. Another three individuals did not report gender. So, analyses were performed with *n* = 313 participants. Another structural equation model was conducted that included gender as a control variable. Therefore, gender as a manifest variable was assumed to impact all five latent variables (warmth, competence, negative emotions, active self-harm, passive self-harm). Because of the centrality of the assumed indirect effects, we also tested whether gender moderated the indirect effects of stereotypes on discrimination *via* prejudice. Independent *t*-tests were chosen because of their robustness testing for differences between women and men in warmth, competence, negative emotions, active self-harm, and passive self-harm.

To show the internalization process, which includes at first considering oneself as belonging to a stigmatized group (i.e., people with mental health problems), we compared the elements of self-stigma between different stages of belonging. These stages should indicate how likely it is that someone considers himself or herself as belonging to the group of people suffering from mental health problems. Five stages were defined: healthy individuals (stage 1, *n* = 393), individuals who passed only the cut-off for alcohol disorders, but did not state to have mental health problems (stage 2, *n* = 93), individuals who passed at least one cut-off for a mental disorder (but not for alcohol disorders) and did not state to have mental health problems (stage 3, *n* = 89), individuals who stated to have mental health problems, but no mental disorder (stage 4, *n* = 46), and individuals who stated to have a mental disorder (stage 5, *n* = 92). We assumed that, based on the relatively young sample that included many college students, those who passed only the cut-off for alcohol disorders but did not state to have mental health problems considered their drinking behavior as non-problematic and normative within a student environment. That implies they would not consider themselves as belonging to those suffering from mental health problems, meaning their self-view is unrelated to self-stigma. Because of this, we defined them as a separate group. One-way ANOVAs were conducted for stereotypes, prejudice, and discrimination, depending on the stage of belonging. Because of violations of assumptions, we used Welch's *F* for testing significance. *Post hoc* tests were performed with the Games–Howell procedure. We also calculated Pearson's correlations between the elements of self-stigma and the number of passed cut-offs of the PHQ-D as another indicator of belonging (subsample A, *n* = 320).

Taking the considerations mentioned above to individuals who passed only the cut-off for alcohol disorders but did not state to have mental health problems into account, we conducted our structural equation models again. This time, the models are based on a sample without those individuals, reducing the sample size to *n* = 227 individuals who suffer from mental health problems.

## Results

### Descriptive Statistics and Intercorrelations

Table 2 shows descriptive statistics and bivariate correlations between all items. The intercorrelations of the latent variables are presented in Table 3. Means and standard deviations of stereotypes (warmth, competence), prejudice, and discrimination (active and passive self-harm) separated for women and men, and testing for differences between them, are shown in Table 4.

**Table 2:** Correlations between mean and standard deviation of all variables (subsample A, *n* = 320).

	**1**	**2**	**3**	**4**	**5**	**6**	**7**	**8**	**9**	**10**	**11**	**12**	**13**	**14**	**15**	**16**
1	1.00															
2	0.44**	1.00														
3	0.29**	0.52**	1.00													
4	0.22**	0.07	0.13*	1.00												
5	0.34**	0.08	0.06	0.47**	1.00											
6	0.44**	0.27**	0.20**	0.44**	0.69**	1.00										
7	−0.44**	−0.12*	−0.12*	−0.34**	−0.34**	−0.38**	1.00									
8	−0.35**	−0.05	−0.14*	−0.28**	−0.30**	−0.36**	0.56**	1.00								
9	−0.32**	−0.09	−0.05	−0.28**	−0.18**	−0.19**	0.49**	0.40**	1.00							
10	−0.44**	−0.11	−0.14*	−0.30**	−0.35**	−0.34**	0.68**	0.60**	0.55**	1.00						
11	−0.27**	−0.07	−0.11*	−0.24**	−0.33**	−0.33**	0.57**	0.40**	0.45**	0.44**	1.00					
12	−0.36**	−0.17**	−0.10	−0.29**	−0.34**	−0.33**	0.59**	0.45**	0.50**	0.51**	0.73**	1.00				
13	−0.29**	−0.11*	−0.13*	−0.23**	−0.25**	−0.24**	0.53**	0.35**	0.41**	0.41**	0.76**	0.72**	1.00			
14	−0.32**	−0.19**	−0.07	−0.25**	−0.26**	−0.23**	0.39**	0.34**	0.35**	0.42**	0.33**	0.42**	0.34**	1.00		
15	−0.29**	−0.21**	−0.10	−0.17**	−0.28**	−0.22**	0.31**	0.31**	0.26**	0.32**	0.31**	0.38**	0.35**	0.66**	1.00	
16	−0.23**	−0.19**	−0.06	−0.18**	−0.17**	−0.17**	0.27**	0.21**	0.25**	0.35**	0.29**	0.31**	0.27**	0.47**	0.43**	1.00
*M*	4.16	4.43	4.45	4.38	3.48	4.01	1.64	2.43	1.09	2.13	0.69	1.04	0.64	2.02	1.81	2.68
*SD*	1.27	1.14	1.13	1.21	1.53	1.33	1.67	1.65	1.50	1.80	1.39	1.57	1.32	1.79	1.71	1.87

1 = likeable, 2 = warm, 3 = good-natured, 4 = independent, 5 = competitive, 6 = competent, 7 = contempt, 8 = anger, 9 = fear, 10 = shame, 11 = active self-harm 1, 12 = active self-harm 2, 13 = active self-harm 3, 14 = passive self-harm 1, 15 = passive self-harm 2, 16 = passive self-harm 3, M = mean, SD = standard deviation, Pearson correlation coefficients, ^*^ p < 0.05 (two-tailed), ^**^ p < 0.01 (two-tailed).

**Table 3:** Correlations between latent variables (Model 1.0, subsample A, *n* = 320). And correct the variable names as follows “Active self-harm”, “Passive self-harm”.

		**1**	**2**	**3**	**4**	**5**
1	Warmth	1.00				
2	Competence	0.41**	1.00			
3	Negative emotions	−0.47**	−0.54**	1.00		
4	Activeself-harm	−0.30**	−0.39**	0.73**	1.00	
5	Passive self-harm	−0.29**	−0.37**	0.58**	0.52**	1.00

Pearson correlation coefficients, ^**^ p < 0.01 (two-tailed).

**Table 4:** Differences between women (*n* = 235) and men (*n* = 78) on stereotypes, prejudice, and discrimination (subsample A, *n* = 320).

	**Women**		**Men**		***t***	***df***	***p***	**Cohen's** ***d***
	***M***	***SD***	***M***	***SD***				
Warmth	4.35	0.93	4.38	0.91	−0.19	311.00	0.850	−0.025
Competence	3.85	1.09	4.30	1.13	−3.12	311.00	0.002	−0.409
Negative emotions	1.93	1.38	1.43	1.10	3.22	165.57	0.002	0.422
Active self-harm	0.93	1.38	0.33	0.84	4.56	219.57	<0.001	0.596
Passive self-harm	2.30	1.51	1.75	1.29	3.13	151.90	0.002	0.409

### Hypothesis Testing

**Figure 1:**
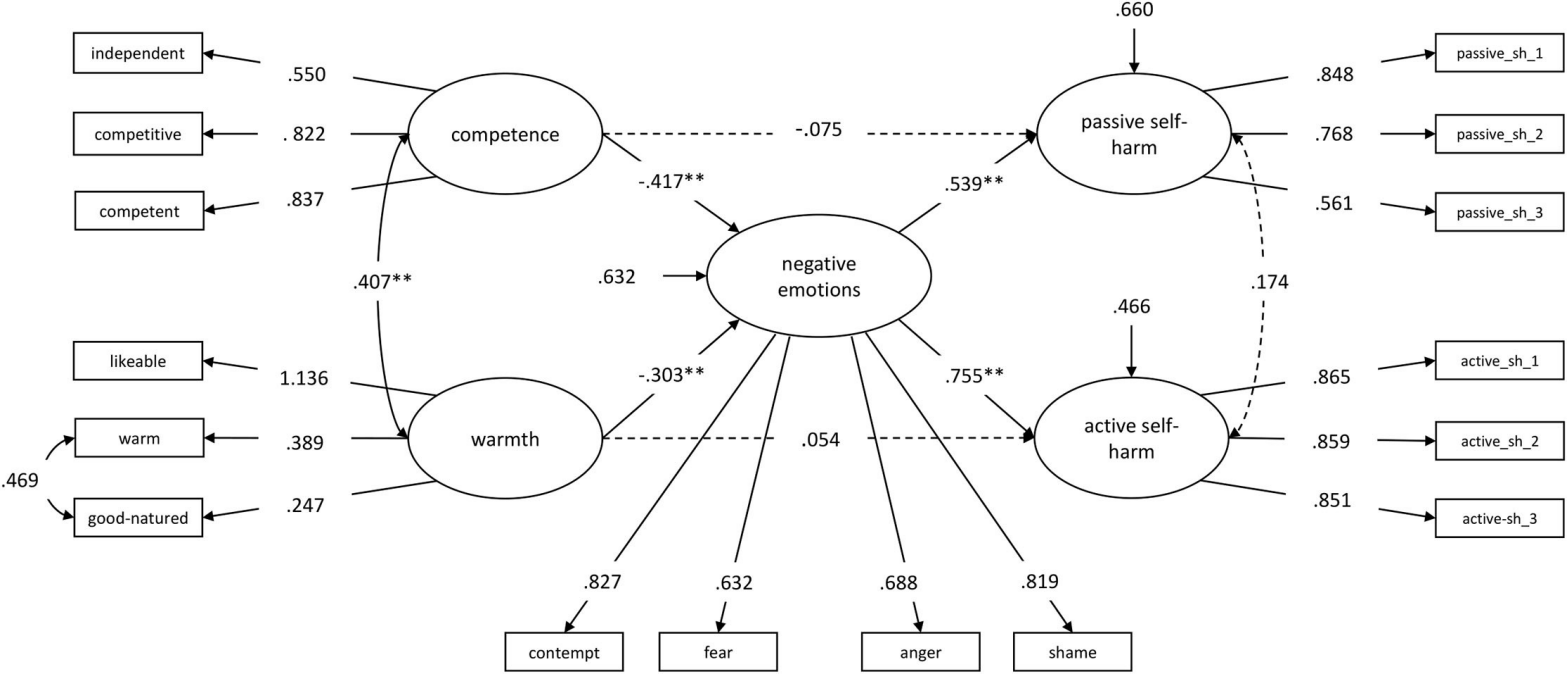
Warmth and competence, predicting passive and active self-harm *via* negative emotions (Model 1.0). Standardized regression weights. Dashed lines are not significant (*p* > 0.05). ^*^ *p* < 0.05, ^**^ *p* < 0.01. sh = self-harm.

We tested our hypotheses regarding the interrelations of stereotypes, prejudice, and discrimination as parts of self-stigma in a structural equation model. The theoretically assumed model (Model 0) figured the latent constructs warmth and competence, including three items each as the manifest variables. Warmth predicted the latent construct active self-harm, competence predicted the latent construct passive self-harm. Active and passive self-harm included three items each as manifest variables. Warmth and competence were allowed to correlate as well as active and passive self-harm. The latent construct negative emotions (prejudice), including four items as manifest variables, were complemented as a mediator. So, warmth predicted active self-harm *via* negative emotions, and competence predicted passive self-harm *via* negative emotions. The manifest variables within the latent constructs were not allowed to correlate. The goodness-of-fit indices were not satisfying for the first model χ^2^(96) = 255.644, *p* < 0.001, *RMSEA* = 0.072 [90% CI = (0.061, 0.083), *CFI* = 0.930, *TLI* = 0.913, *SRMR* = 0.055, *AIC* = 16,373.270, *BIC* = 16,584.296]. Modification indices indicated a correlated residual between the items *warm* and *good-natured* within the latent construct warmth. So, we modified the model and allowed a correlation between these two items. As a result, the Goodness-of-fit indices improved and indicated good model fit χ^2^(95) = 186.196, *p* < 0.001; *RMSEA* = 0.055 [90% CI = (0.043, 0.066)]; *CFI* = 0.960, *TLI* = 0.950, *SRMR* = 0.045, *AIC* = 12,002.207, *BIC* = 12,197.429. Figure 1 shows the standardized regression weights for the model (Model 1.0).

As expected, both warmth [β = −0.303, *B* = −0.309, SE_*B*_ = 0.113, 95% BCI = (−0.543, −0.100), *p* = 0.006] and competence [β = −0.417, *B* = −0.486, SE_*B*_ = 0.095, 95% BCI = (−0.682, −0.308), *p* < 0.001] were negatively related to negative emotions. However, warmth had no direct effect on active self-harm [β = 0.054, *B* = 0.050, SE_*B*_ = 0.054, 95% BCI = (−0.038, 0.180), *p* = 0.348], and competence was not directly related to passive self-harm [β = −0.075, *B* = −0.090, SE_*B*_ = 0.112, 95% BCI = (−0.298, 0.142), *p* = 0.422]. Negative emotions had a positive effect on both, active [β = 0.755, *B* = 0.691, SE_*B*_ = 0.081, 95% BCI = (0.538, 0.857), *p* < 0.001] and passive self-harm [β = 0.539, *B* = 0.556, SE_*B*_ = 0.099, 95% BCI = (0.367, 0.746), *p* < 0.001].

#### Indirect Effects

The theoretically assumed specific indirect effect of competence on passive self-harm *via* negative emotions emerged as significant [β = −0.225, *B* = −0.270, SE_*B*_ = 0.075, 95% BCI = (−0.445, −0.151), *p* < 0.001], as well as the indirect effect of warmth on active self-harm *via* negative emotions [β = −0.228, *B* = −0.214, SE_*B*_ = 0.082, 95% BCI = (−0.399, −0.074), *p* = 0.009]. Furthermore, to our assumptions, we also tested the specific indirect effects of competence on active self-harm *via* negative emotions [β = −0.315, *B* = −0.336, SE_*B*_ = 0.077, 95% BCI = (−0.506, −0.199), *p* < 0.001], as well as the effect from warmth on passive self-harm *via* negative emotions [β = −0.163, *B* = −0.172, SE_*B*_ = 0.072, 95% BCI = (−0.337, −0.056), *p* = 0.016]. Both were significant as well. Negative emotions fully mediated the relationships between the stereotype dimensions (warmth, competence) and discrimination (active self-harm, passive self-harm).

#### Latent Interaction of Warmth and Competence

**Figure 2:**
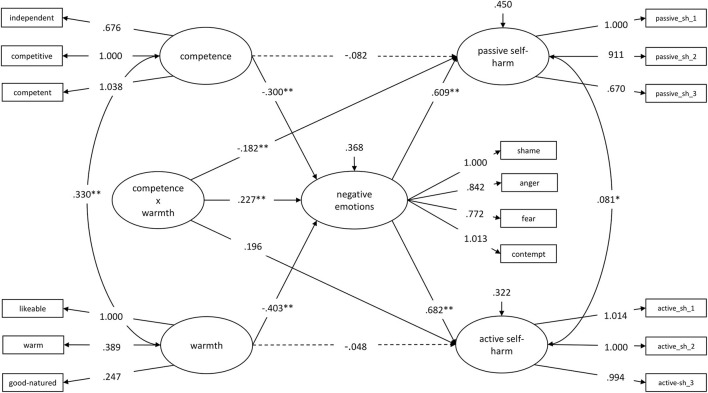
Warmth, competence, and their interaction (warmth x competence), predicting passive and active self-harm *via* negative emotions (Model 2.0). Dashed lines are not significant (*p* > 0.05). * *p* < 0.05, ** *p* < 0.01. sh = self-harm.

Considering the different effects of mixed and consistent stereotypes, we defined another structural equation model with a latent interaction term of warmth x competence. Model 0 was the foundation for this. It was complemented by the interaction of warmth x competence, which predicted negative emotions, passive self-harm, and active self-harm. Figure 2 shows the regression weights of Model 2.0. The relatively lower information criterions (*AIC* = 12,417.358, *BIC* = 12,639.689), and the result of the log-likelihood ratio test [*D*_(3)_ = 3961.912, *p* < 0.001] indicated better model fit for Model 2.0 compared to Model 0.

The conditional indirect effects of competence on passive self-harm *via* negative emotions emerged significant for those low in warmth [1 *SD* below *M*_Warmth_; *B* = −0.321, SE_*B*_ = 0.079, 95% CI = (−0.476, −0.166), *p* < 0.001] and for those with medium-warmth levels [*M*_Warmth_; *B* = −0.183, SE_*B*_ = 0.052, 95% CI = (−0.286, −0.080], *p* < 0.001], but not for those high in warmth {1 *SD* above *M*_Warmth_; *B* = −0.044, SE_*B*_ = 0.065, 95% CI = [−0.173, 0.084], *p* = 0.496; s. (Supplementary Figure 1)}. The conditional indirect effect of warmth on active self-harm *via* negative emotions was significant for those low in competence [1 *SD* below *M*_Competence_; *B* = −0.430, SE_*B*_ = 0.088, 95% CI = (−0.601, −0.258), *p* < 0.001] and for those with medium-competence levels [*M*_Competence_ *B* = −0.275, SE_*B*_ = 0.064, 95% CI = (−0.399, −0.150), *p* < 0.001], but not for those high in competence [1 *SD* above *M*_Competence_; *B* = −0.120, SE_*B*_ = 0.076, 95% CI = (−0.269, 0.029), *p* = 0.116; (Supplementary Figure 2)]. The effect from competence on negative emotions was significant for those low on warmth [1 *SD* below *M*_W_; *B* = −0.528, SE_*B*_ = 0.107, 95% CI = (−0.737, −0.319), *p* < 0.001] and for those with medium warmth [*M*_Warmth_; *B* = −0.300, SE_*B*_ = 0.076, 95% CI = (−0.450, −0.151), *p* < 0.00], but not for those high on warmth [1 *SD* above *M*_W_; *B* = −0.073, SE_*B*_ = 0.107, 95% CI = (−0.283, 0.137), *p* = 0.496; (Supplementary Figure 3)]. Similar relations were found for the effect from warmth on negative emotions, depending on competence [low: *B* = −0.630, SE_*B*_ = 0.115, 95% CI = (−0.856, −0.404), *p* < 0.001; medium: *B* = −0.403, SE_*B*_ = 0.083, 95% CI = (− 0.565, −0.240), *p* < 0.001; high: *B* = −0.175, SE_*B*_ = 0.108, 95% CI = (−0.388, 0.037), *p* = 0.106; (Supplementary Figure 4)].

#### Additional Analyses

Considering gender differences, women perceived themselves less competent and experienced more negative emotions as well as active and passive self-harm compared to men (see Table 4). Referring to those differences, gender (1 = women, 2 = men) was then added as a control variable in Model 1.0, with impact on all five latent variables. Model fit declined (χ^2^(107) = 258.113, *p* < 0.001, *RMSEA* = 0.067 [90% CI = (0.057, 0.078), *CFI* = 0.933, *TLI* = 0.915, *SRMR* = 0.082], while gender had a significant impact on competence [β = 0.215, *B* = 0.651, SE_*B*_ = 0.207, 95% CI = (0.253, 1.057), *p* = 0.002], warmth [β = 0.091, *B* = 0.454, SE_*B*_ = 0.173, 95% CI = (0.118, 0.784), *p* = 0.008] and active self-harm [β = −0.091, *B* = −0.275, SE_*B*_ = 0.109, 95% CI = (−0.480, −0.053), *p* = 0.011]. A conditional indirect effect of gender was neither found for the effect from competence on passive self-harm *via* negative emotions nor for warmth on active self-harm *via* negative emotions.

**Figure 3:**
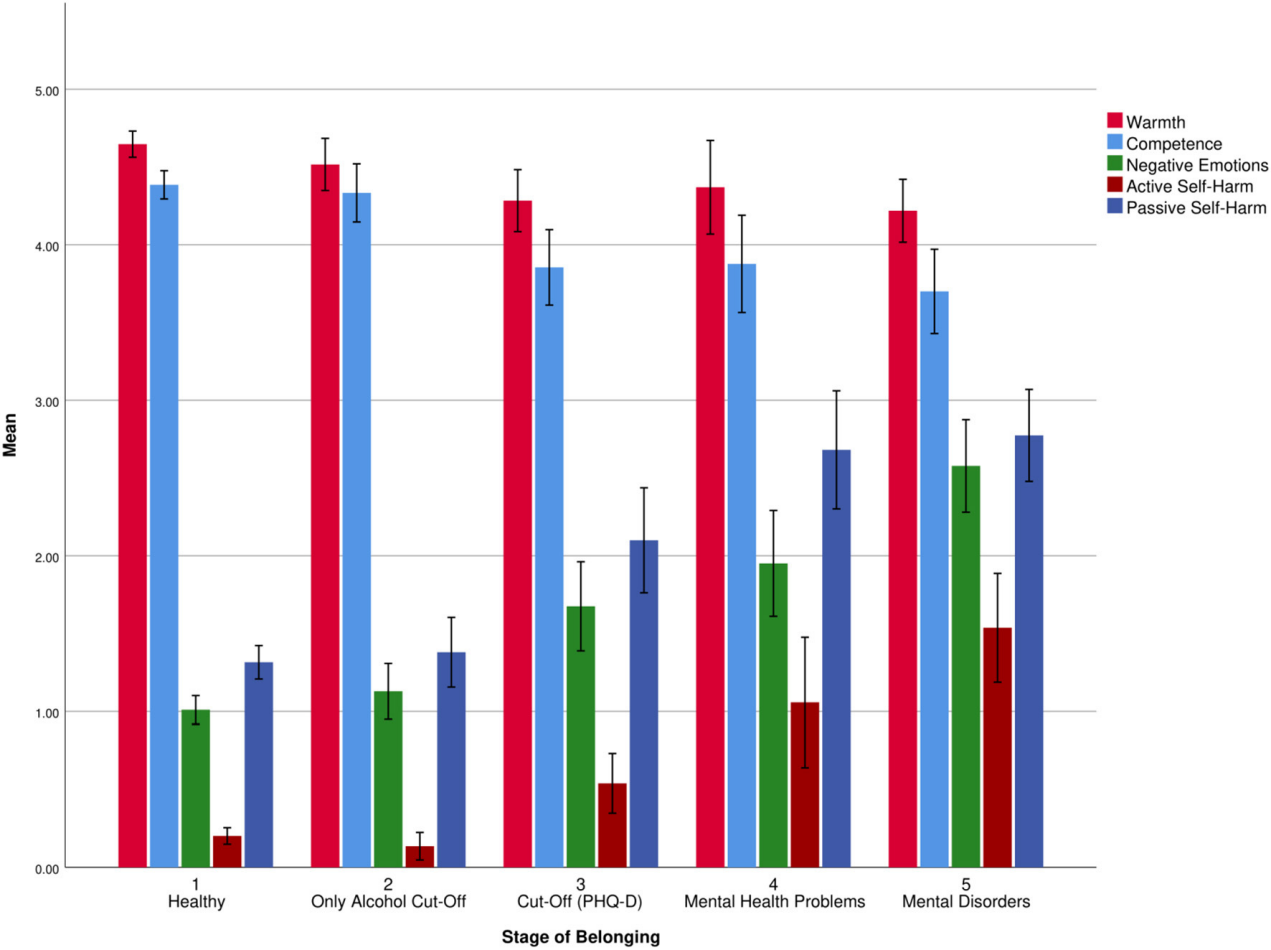
Means and error bars (95% CI) of warmth, competence, negative emotions, active self-harm, and passive self-harm for each stage of belonging, referring to the process of internalization.

Figure 3 shows means of warmth, competence, negative emotions, active self-harm, and passive self-harm for each stage of belonging to those suffering from mental health problems. We tested the internalization process and found significant differences between the different stages of belonging for warmth [*F*(4, 173.214) = 5.811, *p* < 0.001], competence [*F*(4, 170.899) = 10.632, *p* < 0.001], negative emotions [*F*(4, 167.158) = 32.676, *p* < 0.001], active self-harm [*F*(4, 163.166) = 22.381, *p* < 0.001], and passive self-harm [*F*(4, 169.767) = 33.787, *p* < 0.001]. Based on *post hoc* analyses, individuals who reported mental disease (Stage 5) perceived themselves less warm (*M*_Diff_ = −0.42, *p* < 0.001, 95% CI (−0.71, −0.12)] and less competent than healthy individuals [Stage 1; *M*_Diff_ = −0.69, *p* < 0.001, 95% CI (−1.08, −0.30)]. The same was found for those who passed at least one cut-off of the PHQ-D [Stage 3; warmth: *M*_Diff_ = −0.35, *p* = 0.01, 95% CI (−0.65, −0.06); competence: *M*_Diff_ = −0.53, *p* < 0.001, 95% CI (−0.88, −0.20)] compared to healthy ones. There was no significant difference of warmth and competence between Stage 3 and 5. Individuals, who reported mental disorders (Stage 5), perceived more negative emotions [*M*_Diff_ = 0.90, *p* < 0.001, 95% CI (0.33, 1.48)] and showed more active [*M*_Diff_ = 1.03, *p* < 0.001, 95% CI (0.66, 1.39)] and passive self-harm [*M*_Diff_ = 0.66, *p* = 0.03, 95% CI (0.04, 1.27)] than those who passed at least one cut-off of the PHQ-D without reporting mental health problems (Stage 3). The number of passed cut-offs for mental disorders was significantly correlated with warmth {*r*(318) = −0.20, *p* < 0.001), competence [*r*(318) = −0.32, *p* < 0.001]}, negative emotions [*r*(318) = 0.31, *p* < 0.001], active self-harm [*r*(318) = 0.36, *p* < 0.001], and passive self-harm [*r*(318) = 0.33, *p* < 0.001].

Finally, we ran Model 1.0 and Model 2.0 once again without those participants, who only passed the cut-off for alcohol disorder of the PHQ-D but did not state to have mental health problems. Reducing the sample size to *n* = 227, Model 1.1 showed still good model fit (χ^2^(95) = 143.850, *p* = 0.001; *RMSEA* = 0.048 [90% CI = (0.031, 0.063)]; *CFI* = 0.969, *TLI* = 0.960, *SRMR* = 0.049, *AIC* = 12002.207, *BIC* = 12197.429. Model 2.1 (*AIC* = 8901.697, *BIC* = 9103.769) improved compared to Model 2.0.

## Discussion

This study aimed to apply the SCM (Fiske et al., 2002) and the BIAS map (Cuddy et al., 2007) to self-stigma among people with mental health problems by examining the effects of stereotypes (warmth, competence) on discrimination (active self-harm, passive self-harm) *via* prejudice (negative emotions). In line with the predictions of the SCM, it was found that both stereotype dimensions warmth and competence were negatively related to negative emotions. Low warmth, as well as low competence, was associated with more negative emotions, while these relations were even stronger when the other dimension was low, too. Furthermore, more negative emotions were associated with higher amounts of active and passive self-harm. Warmth and competence had no direct effect on active and passive self-harm. Negative emotions fully mediated the relationship between warmth and active self-harm as well as between competence and passive self-harm. Considering the interaction of the stereotype dimensions, the indirect negative effects of warmth and competence on active and passive self-harm *via* negative emotions were stronger when competence and warmth were low, respectively. Analyses also indicated indirect effects between warmth and passive self-harm plus competence and active self-harm. Based on the simultaneous observation of warmth and competence, it can be assumed that high values on one dimension protect against the negative impact of low values on the other dimension. These findings are consistent with the theoretical framework of the SCM (Fiske et al., 2002) and the BIAS map (Cuddy et al., 2007). Emotions affect behavior tendencies more strongly than stereotypes, emotions mediate the link between stereotypes and behavior tendencies and mixed and consistent stereotypes predict distinct emotional and behavioral reaction. Additionally, gender seems to impact self-stigma inasmuch as men see themselves more competent and experience less active self-harm than women. Considering oneself to belong to a stigmatized group is an important condition when self-stigma is internalized.

### Why We Should Use the SCM and the BIAS Map Framework to Examine Self-Stigma

Previous research has already explored single elements of self-stigma: Typical stereotypes about the self, for example, incompetence and dangerousness (Corrigan et al., 2011; Corrigan and Rao, 2012), are related to emotions like fear and shame, which are conceptualized as prejudice (Rüsch et al., 2010; Corrigan and Rao, 2012; Hasson-Ohayon et al., 2012), while discrimination is featured by self-isolation (Corrigan and Rao, 2012). However, these stigma components were often examined separately or were operationalized differently based on various theoretical backgrounds, even though they try to describe elements influencing each other within self-stigma. Our study addressed these limitations by encompassing all three elements of self-stigma within one theoretical framework. The SCM and the BIAS map are well established and empirically confirmed (Fiske and Durante, 2016; Fiske, 2018). In comparison to previous theoretical approaches, they have the advantages of making more systematical predictions and strengthening the relationship between the stereotype dimensions warmth and competence, emotions, and discrimination within one theoretical framework. Using the SCM and the BIAS map to examine self-stigma seems appropriate because these models contain the same three elements as self-stigma: stereotypes, prejudice, and discrimination. By applying the SCM and the BIAS map to self-stigma, we were able to observe the single components of self-stigma as such and examine the relationships between all the components. By doing that, we could demonstrate that the predictions from the SCM and the BIAS map are appropriate within the concept of self-stigma. Overall, warmth and competence within the SCM and the BIAS map framework seem to be suitable to analyze both—public and self-stigma of mental illness. The applicability to public stigma has already been shown (Sadler et al., 2015; Boysen, 2017). So, it seemed deducible to transfer these findings on public stigma to self-stigma, because both share fundamental contents: Since, within both concepts, stereotypes relate to incompetence or dangerousness in terms of poor warmth (Parcesepe and Cabassa, 2013), prejudice to fear, anger, or pity (Corrigan et al., 2003; Angermeyer et al., 2010), and discrimination to social distancing or withholding help (Corrigan, 2000; Parcesepe and Cabassa, 2013).

### Clinical Implications

The SCM and the BIAS map framework allows, based on the characteristics of warmth and competence, to make more specific predictions about a person's likely behavioral reaction. For example, a person who perceives himself or herself as highly incompetent and moderately warm is more likely to show passive self-harming behavior than active self-harming behavior. And a behavioral reaction will be even stronger by simultaneous high activation of emotions like contempt or shame. Moreover, a greater extent of one stereotype dimension seems to protect against increased negative emotions or self-harming caused by a low extent of the other stereotype dimension. This could be relevant for risk prediction or planning effective clinical interventions. A further advantage is the fundamentality of warmth and competence (Fiske et al., 2002) because it seems promising to integrate findings from related theories like attribution theory (Corrigan et al., 2003) or intergroup contact (Kotzur et al., 2019) so that they can be applied to self-stigma as well. The SCM and the BIAS map seem to be suitable for describing self-stigma among individuals with mental health problems and allowing us to deepen our knowledge of the specific interactions between the elements of self-stigma. A better understanding of self-stigma based on well-established and empirically confirmed theories (such as the SCM and the BIAS map) may help elucidate critical mechanisms. Shedding more light on these mechanisms by experimental manipulation or longitudinal studies may help develop powerful self-stigma interventions, aiming to increase the quality of life and help-seeking behavior and reduce depressiveness, suicidality, and self-harm. Self-stigma, including self-harm, is prevalent among various mental disorders (Griffiths et al., 2015; Bonfils et al., 2018; Dubreucq et al., 2020). As a result, self-harm could be a relevant psychopathology in the wake of self-stigma, even for mental disease whose diagnostic criteria do not include self-harm as a symptom (which may be a reason why self-harm is often overlooked). A deeper understanding of self-stigma would allow to identify those patients and treat them with targeted interventions. Furthermore, it is reasonable to conjecture that self-harm resulting from self-stigma has to be treated differently than self-harm in consequence of emotional dysregulation or dissociation.

### Limitations and Future Research

The current research used the same items to measure the stereotype dimensions warmth and competence as it is common practice in the SCM intergroup or interindividual research. However, the items measuring warmth did not indicate high loadings on the same factor. Instead, the strong relationship between the items *warm* and *good-natured* makes it conceivable that warmth, when referring to the self, may be better described by two facets: sociability and morality (Brambilla and Leach, 2014). Morality refers to perceived correctness, while sociability concerns cooperation and forming connections (Leach et al., 2007; Brambilla et al., 2011). At the group level, morality seems more critical than sociability (Leach et al., 2007; Brambilla et al., 2011, 2019), but, maybe, morality loses its dominance when it comes to the self-perspective (Brambilla and Leach, 2014). That would imply that it is more important for an individual to perceive oneself as being able to cooperate and form connections with others than acting morally. Future research should consider this by using items that represent both—sociability and morality. This may help to examine which component is more important for the self. While transferring assumptions from the intergroup and interindividual perspective to the self-perspective, the values of warmth and competence should be considered as well. Abele and Wojciszke (2014) refer to communal and agentic content as fundamental dimensions in their *Dual Perspective Model* (DPM-AC), which are similar to warmth and competence as fundamental dimensions in the SCM (Fiske et al., 2002) and the BIAS map (Cuddy et al., 2007). They could show that communion (warmth) and agency (competence) differ in their values, depending on the perspective. That means that agency is more important in the self-perception (intraindividual context) while communion is more meaningful in the other perception (intergroup/interindividual context; Abele et al., 2021). So, the question arises whether competence is more important than warmth regarding self-stigma. Our findings were not able to answer this question. The interaction of warmth and competence seems to play an important role while making specific predictions for emotional and behavioral reactions. Thus, it is also conceivable that the distinction between warmth and competence is not important for self-stigma-affected people. While internalizing negative stereotypes from society, the other perspective is addressed and warmth is given more weight. Simultaneously, the self is evaluated on focuses of competence. In the end, the values of warmth and competence compensate for each other and are of equal importance. This could explain why self-stigma is resulting not only in one type of self-harming behavior. Future research should pursue the question whether one stereotype dimension is more important than the other or whether the relation between both dimensions is crucial while predicting self-harm. This is highly relevant concerning the severity of self-harming behavior. Individual differences like gender or personality traits are further issues that future research on self-stigma should address. Our findings that men perceive themselves as more competent than women go along with research on the SCM, demonstrating that typical men are seen as highly competent and typical women as low competent (Eckes, 2002; Fiske, 2010). In line with this, our findings suggest that men, overall, experience less self-stigma. However, especially because men are seen as highly competent, some research suggests that this is why they are experiencing even more self-stigma (Latalova et al., 2014). Other research showed that women are suffering more from self-stigma, and, still, others found no gender difference (Evans-Lacko et al., 2012; Shimotsu and Horikawa, 2016; Kalisova et al., 2018; Mackenzie et al., 2019). So, current evidence of gender and self-stigma remains unclear. A pretty similar picture can be drawn for the relationship between personality traits and self-stigma. While some research indicated evidence for the association between certain personality traits [e.g., avoidant traits, self-directedness (Ociskova et al., 2015; Dubreucq et al., 2021) and self-stigma, others did not (Ingram et al., 2016)]. Thus, this seems to be an important issue with mixed evidence that also needs further research. By taking the cross-sectional study design into account, the observed relationships between stereotypes, prejudice, and discrimination or, more precisely, the indirect effects of warmth and competence on active and passive self-harm *via* negative emotions are only based on theoretical assumptions. In other words, we were not able to test causality. Nevertheless, experimental research supports the generic assumption that emotions mediate the relation between stereotypes and discrimination like it is predicted by the SCM and the BIAS map (Caprariello et al., 2009; Echebarria-Echabe, 2013; Kotzur et al., 2019; Sevillano and Fiske, 2019). Based on this, it seems promising to approve the cross-sectional relations in an experimental paradigm applied to self-stigma as well. In view of the key position of emotions in the self-stigma process, our findings are well integrable. We found that negative emotions (prejudice) mediate the relationship between the stereotype dimensions (warmth, competence) and discriminating behavior (active/passive self-harm). Here, prejudice includes different emotions (*contempt, shame, anger, fear*) from the low competence/low warmth cluster. The high internal consistency (Cronbach's α = 0.83), as well as the high loadings on the latent factor *negative emotions*, supports the assumption that these four items form one cluster so that it is adequate to conflate them. However, future research should consider these emotions separately as well. This would allow to observe which emotion out of the low competence/low warmth cluster enhances self-harming behavior the most. This could be helpful when thinking about specific interventions to reduce self-stigma. All participants completed the survey in the same order. Completing measures related to one's mental health may have primed the negative perceptions of mental health challenges and led to higher intercorrelations of the stereotype, emotion, and behavior measures. This should be kept in mind when interpreting the results. Mental health status was assessed by self-report using a screening instrument. Diagnoses have not been confirmed by professionals, making it difficult to compare the results to other research addressing people with mental illness or even inpatients. At the same time, it can be assumed that self-stigma would be more pronounced in a population of people with severe mental illnesses, resulting in lower scores of warmth and competence as well as stronger prejudice and self-harming behavior; thus, self-stigma would be even more relevant. Furthermore, the sample included people with different diagnoses, forming a heterogenous group, which allows only generic conclusions. Future research should target specific diagnoses and make sure that all participants have a confirmed diagnosis. Thus, it would be more clear which patients suffer from self-stigma to what extent.

## Conclusion

This is the first study using the SCM and the BIAS map to describe self-stigma among people with mental health problems. In line with the theoretical assumptions, results indicated that the stereotype dimensions competence and warmth were associated with prejudice (negative emotions), which resulted in passive and active self-harm, while prejudice (negative emotions) fully mediated the relationship between stereotypes and discrimination. This is the first indication for the appropriate application of the SCM and the BIAS map to self-stigma and their suitability to explain public stigma and self-stigma among people with mental health problems. A more detailed understanding of the self-stigma of mental illness based on the specific and accurate predictions from the SCM and the BIAS map could be highly relevant in clinical practice and hopefully helps to reduce the negative outcomes of self-stigma, for example, suicidality (Oexle et al., 2018), non-adherence or dropout from treatment (Corrigan et al., 2014). Future studies should experimentally manipulate single elements of self-stigma or conduct longitudinal studies to further test the assumptions of the SCM and the BIAS map in self-stigma. However, more research is needed to shed more light on all of this.

## Data Availability Statement

The raw data supporting the conclusions of this article will be made available by the authors, without undue reservation.

## Ethics Statement

The studies involving human participants were reviewed and approved by Local Ethics Committee, Department of Psychology at Philipps University of Marburg. The patients/participants provided their written informed consent to participate in this study.

## Author Contributions

LG and SS contributed to the conception and design of the study. LG collected the data and wrote the first draft of the manuscript. LG, FA, FE, and SS analyzed the data and interpreted the results. All authors contributed to manuscript revision, read, and approved the submitted version.

## Funding

Open Access funding provided by the Open Acess Publication Fund of Philipps-Universität Marburg with support of the Deutsche Forschungsgemeinschaft (DFG, German Research Foundation).

## Conflict of Interest

The authors declare that the research was conducted in the absence of any commercial or financial relationships that could be construed as a potential conflict of interest.

## Publisher's Note

All claims expressed in this article are solely those of the authors and do not necessarily represent those of their affiliated organizations, or those of the publisher, the editors and the reviewers. Any product that may be evaluated in this article, or claim that may be made by its manufacturer, is not guaranteed or endorsed by the publisher.

## Footnotes

^1^German wording: sympathisch, warmherzig, gutmütig, eigenständig, konkurrenzfähig, kompetent.

^2^German wording: Verachtung, Wut, Angst, Scham.

^3^Abbreviations refer to Figure 1.

^4^German wording: “Gesund oder krank? Wann helfen wir anderen und uns selbst? Online-Studie zur gesundheitsbezogenen Selbst- und Fremdwahrnehmung”.

## Supplementary Material

Supplementary Figure 1 Indirect effect of competence on passive self-harm *via* negative emotions moderated by warmth. *M* = Mean.

Supplementary Figure 2 Indirect effect of warmth on active self-harm *via* negative emotions moderated by competence. *M* = mean.

Supplementary Figure 3 Effect of competence on negative emotions moderated by warmth. *M* = mean.

Supplementary Figure 4 Effect of warmth on negative emotions moderated by competence. *M* = Mean.
