# Supplementary figures and images for: Self-Stigma Among People With Mental Health Problems in Terms of Warmth and Competence
Source: Front Psychol. 2022 Jun 14;13:877491. doi: 10.3389/fpsyg.2022.877491 (PMC9237425; doi:10.3389/fpsyg.2022.877491)

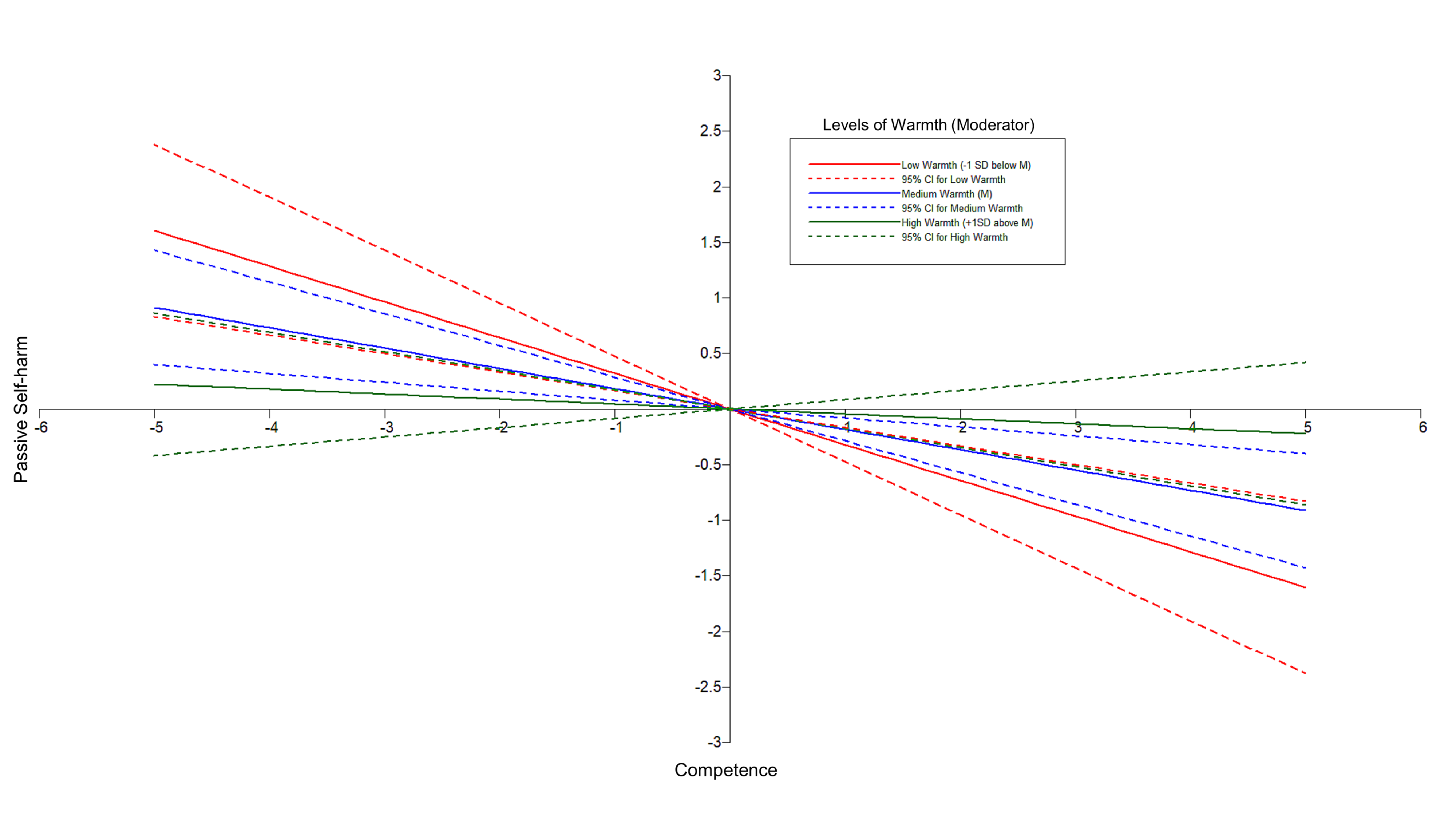

Supplement: Supplementary Figure 1 — Indirect effect of competence on passive self-harm via negative emotions moderated by warmth. M = Mean. [file Image_1.TIFF]

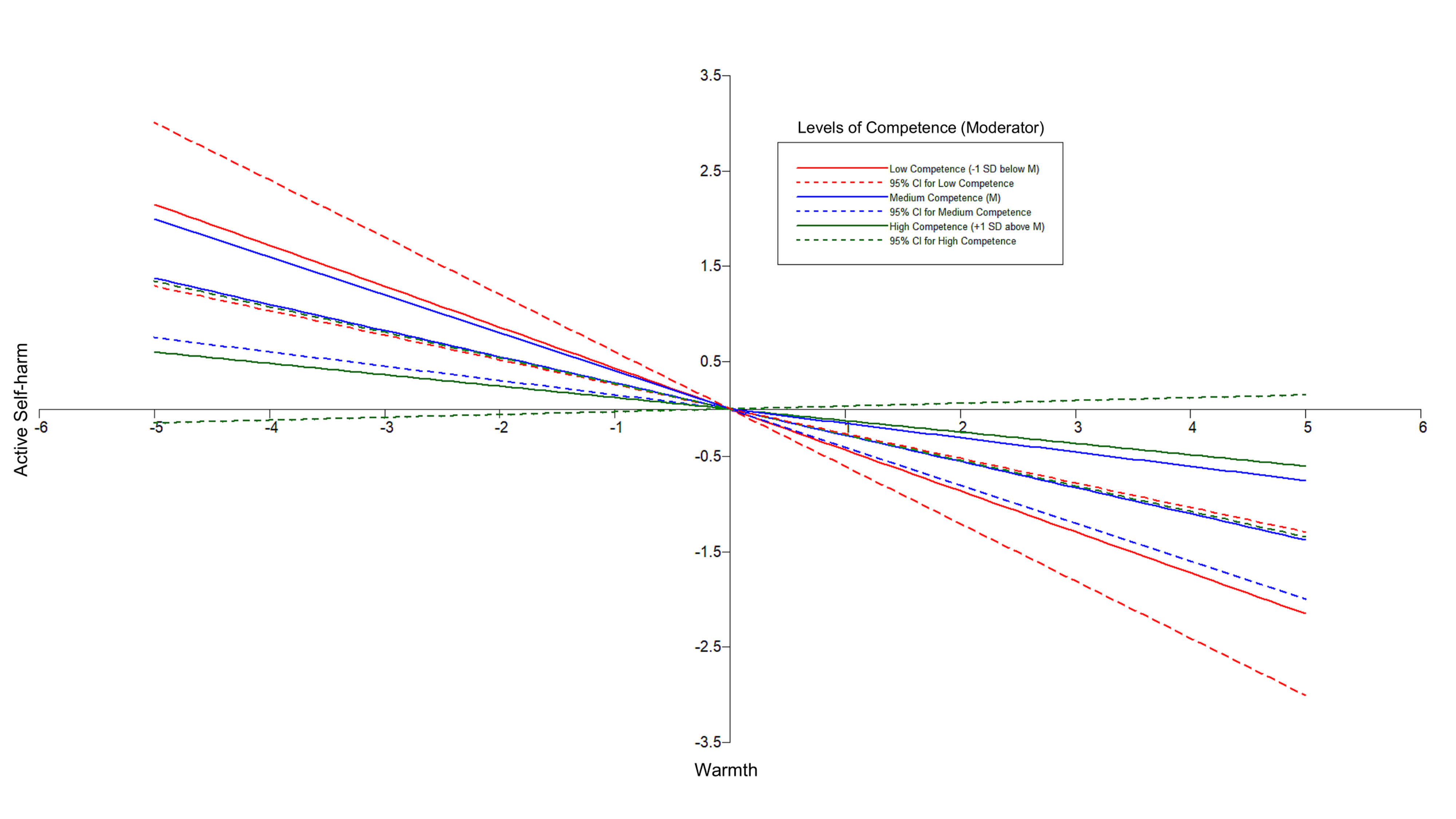

Supplement: Supplementary Figure 2 — Indirect effect of warmth on active self-harm via negative emotions moderated by competence. M = mean. [file Image_2.TIFF]

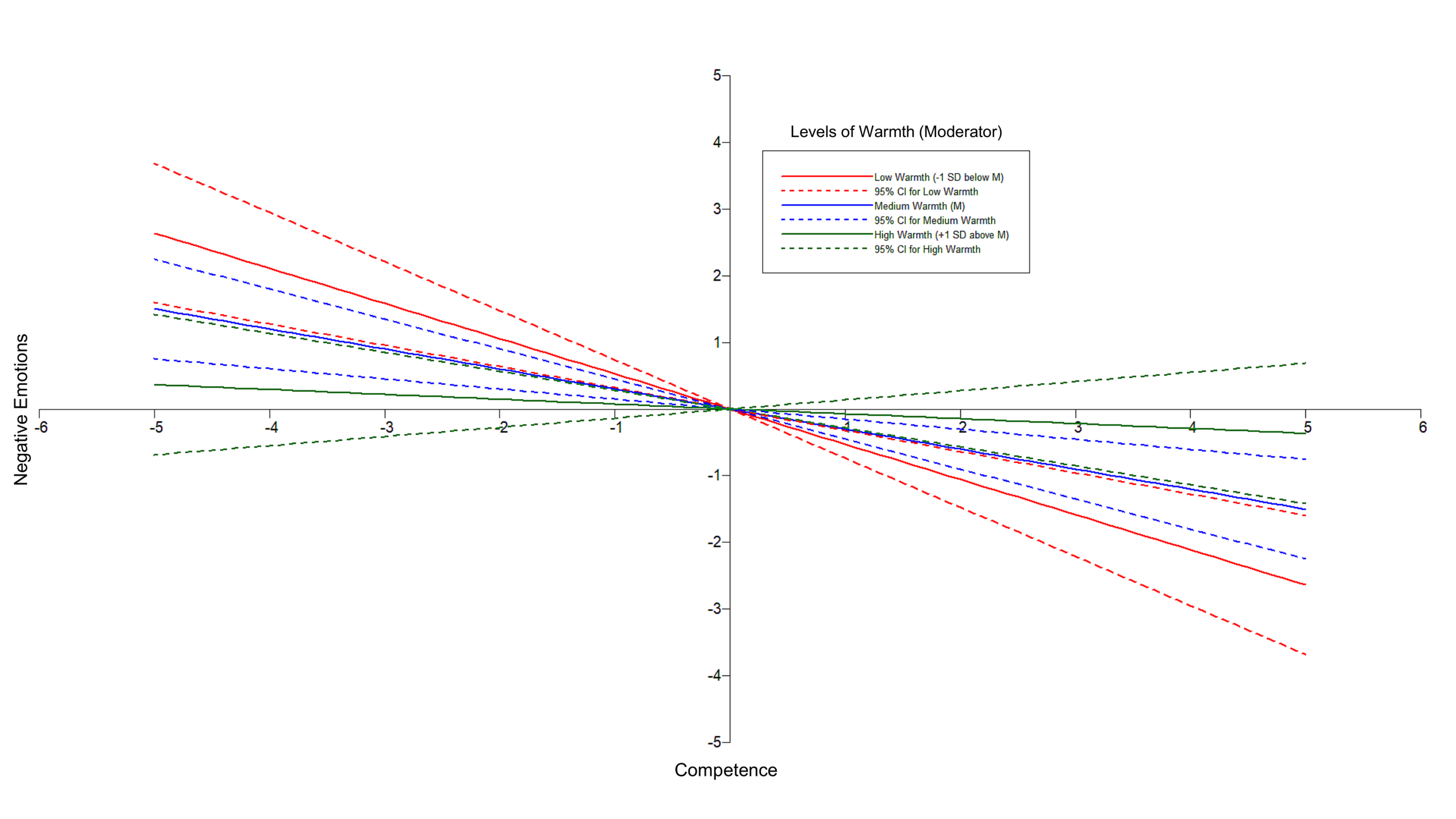

Supplement: Supplementary Figure 3 — Effect of competence on negative emotions moderated by warmth. M = mean. [file Image_3.TIFF]

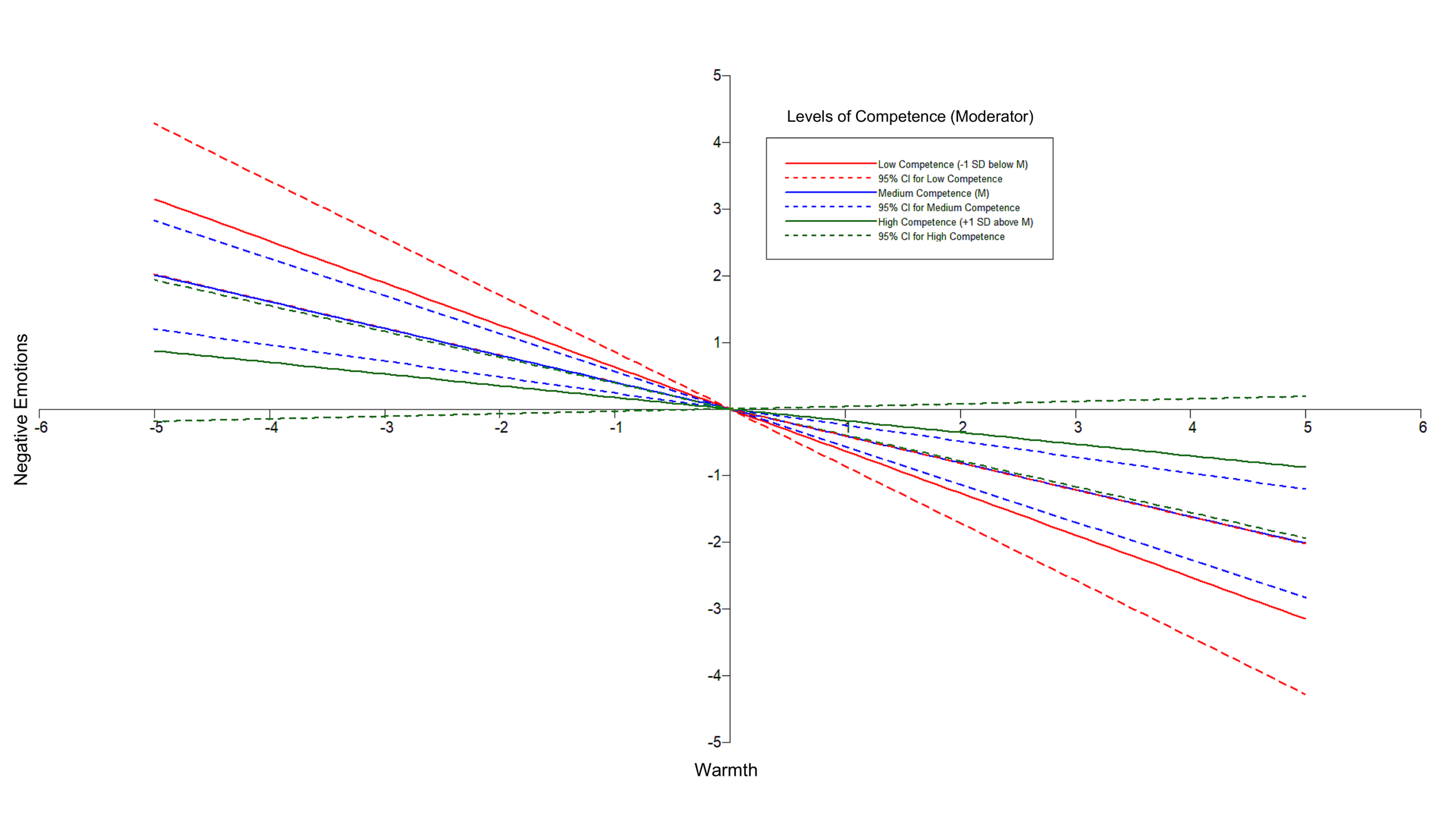

Supplement: Supplementary Figure 4 — Effect of warmth on negative emotions moderated by competence. M = Mean. [file Image_4.TIFF]
